# Clinical Trial Landscape of Duchenne Muscular Dystrophy in Europe: A 10-Year Retrospective Analysis

**DOI:** 10.3390/medicina62101921

**Published:** 2026-10-05

**Authors:** Petar Telbiyski, Radiana Staynova

**Affiliations:** 1 CLINECA AD, 4002 Plovdiv, Bulgaria; 2 Department of Organisation and Economics of Pharmacy, Faculty of Pharmacy, Medical University of Plovdiv, 4002 Plovdiv, Bulgaria

**Keywords:** Duchenne muscular dystrophy, clinical trials, rare diseases, paediatric drug development, clinical trial registries, EudraCT, Clinical Trials Information System (CTIS), Europe

## Abstract

*Background and Objectives*: Duchenne muscular dystrophy (DMD) is a rare and progressive genetic disease with limited treatment options. Clinical trials remain essential for the development of new therapies and may provide patients with access to new potential treatments. This study aims to describe the clinical trial landscape for DMD therapies in the European Union and European Economic Area (EU/EEA) between 2016 and 2026. *Materials and Methods*: A retrospective descriptive analysis was conducted using publicly available data from EudraCT and the Clinical Trials Information System (CTIS). Interventional clinical trials investigating medicinal products for DMD were included if they were active between January 2016 and June 2026 and had at least one investigational site in an EU/EEA country. *Results*: The search identified 95 clinical trial records, of which 80 remained after deduplication, and 62 met the inclusion criteria. Phase III trials accounted for 37.1% (*n* = 23), followed by Phase II trials at 30.6% (*n* = 19). At the time of data extraction, 19 trials had been completed, 17 were ongoing, and 19 had been terminated prematurely. Five trials had been authorised but had not yet started recruitment, while two were temporarily halted due to safety concerns. Trial initiation varied between years, but no consistent trend was identified. Geographic participation was uneven, with the highest activity recorded in Italy, Spain, Belgium, and France. *Conclusions*: No consistent temporal trend in DMD trial initiation was observed during the study period. Most included trials were conducted in Phase II or III. However, the high number of terminated studies and the concentration of trial activity in a limited number of countries show that scientific, operational, and geographic challenges remain.

## 1. Introduction

Duchenne muscular dystrophy (DMD) is a severe genetic disorder leading to a progressive loss of function, primarily affecting young boys [1]. It is the most common form of muscular dystrophy in children, affecting 1 in 3500–5000 live male births [2]. The disease is caused by a mutation in the dystrophin gene, resulting in reduced or absent production of functional dystrophin. Dystrophin is a crucial protein for the structure of muscle cells, and it maintains the integrity of muscle cell membranes [3]. The deficiency of functional dystrophin leads to muscle damage, chronic inflammation, progressive muscle degeneration, and replacement of muscle tissue by fibrosis [1]. Clinically, patients with DMD experience progressive muscle weakness, declining mobility, and loss of ambulation. In the later stages of the disease, respiratory and cardiac muscles are damaged, followed by severe complications, including respiratory insufficiency and cardiomyopathy, which are the primary causes of morbidity and mortality in DMD patients [4].

Despite advances in clinical development, therapeutic options for DMD in Europe remain limited. Only a small number of therapies have been approved by the European Medicines Agency (EMA), including vamorolone and givinostat as more recent agents, and ataluren, for which the conditional marketing authorisation was not renewed in March 2025 [5,6,7]. As a result, access to new therapies is restricted, and substantial medical need persists. In this context, participation in clinical trials represents an important pathway for accessing emerging and innovative therapies.

### 1.1. Clinical Trial Regulation in the EU/EEA

Clinical trials within the European Union and European Economic Area (EU/EEA) are conducted under a harmonised regulatory framework based on European Union (EU) legislation. Directive 2001/20/EC introduced common requirements for the conduct of clinical trials of medicinal products for human use and led to the establishment of EudraCT as the clinical trials database [8,9]. However, because the Directive was implemented through national legislation, differences between Member States created administrative complexity, particularly for multinational clinical trials [10]. The current regulatory framework is established in Regulation (EU) No. 536/2014, which replaced the previous Directive 2001/20/EC and introduced a centralised process for the submission, assessment, supervision and public registration of clinical trials across Member States [11,12].

### 1.2. Clinical Trial Databases in the EU/EEA

Publicly available clinical trial registries play a central role in improving access to clinical research data worldwide. The main European databases developed and maintained by the EMA are European Union Drug Regulating Authorities Clinical Trial Database (EudraCT) and the Clinical Trials Information System (CTIS) [9,12]. Both databases represent different regulatory frameworks and time periods [8,11]. Together, these two registries enable a comprehensive analysis of clinical trial activity over time.

EudraCT was introduced in 2004 under Directive 2001/20/EC as a registry for all interventional trials conducted in the EU/EEA [9]. In addition to EU/EEA trials, EudraCT also includes certain trials conducted outside the EEA that are part of a Paediatric Investigation Plan (PIP) and/or are conducted under Article 45 or 46 of Regulation (EC) No. 1901/2006 [9,13]. The public portal, EU Clinical Trials Register, provides access to selected protocol and results information from EudraCT, including trial design, sponsor, investigational products, inclusion and exclusion criteria, trial status, and participating countries [9]. The availability of results in EudraCT has been inconsistent, particularly for older studies completed before the mandatory results reporting requirements, which were introduced in 2014 [14].

CTIS started operating on 31 January 2022 following the implementation of Regulation (EU) No 536/2014 and gradually replaced EudraCT as the main submission platform for clinical trials in the EU/EEA, becoming mandatory for all new clinical trial applications from 31 January 2023 [12]. CTIS is used as a single system for submission, assessment, authorisation, supervision and public registration of clinical trials. Its public portal provides information on trial design, sponsors, investigational medicinal products (IMPs), recruitment status, and information on sites and points of contact. Compared with the previous framework, CTIS was designed to improve harmonisation, increase transparency, and facilitate the conduct of multinational trials across the EU/EEA [15].

The aim of this study is to analyse the clinical research activity in DMD over the last decade in the EU/EEA by characterising trends in trial design and development stages for new therapies, and to evaluate key variables available in the EU clinical trial registries.

## 2. Materials and Methods

A retrospective descriptive analysis of clinical trials investigating medicinal products for DMD was conducted using publicly available data from the CTIS and EudraCT. A systematic search using the keywords “DMD” and “Duchenne muscular dystrophy” entered separately into the free-text search function for each registry was performed. No additional filters related to trial phase, study status, sponsor, age group, or country were used during the initial search. All retrieved records were manually screened against the predefined eligibility criteria to identify potential studies for inclusion. All interventional trials active within the EU/EEA between January 2016 and June 2026 were considered for inclusion.

Trials were included in the analysis if they (1) were active during the predefined study period, including trials initiated before 2016 but ongoing after January 2016; (2) evaluated an IMP for DMD; (3) were conducted in at least one EU Member State or in Iceland, Liechtenstein, or Norway, the three non-EU countries of the EEA; and (4) contained sufficient publicly available information on the study in the database to allow extraction of key variables. The database search and extraction were completed in June 2026 (Table S1–S3).

The geographic eligibility criteria were based exclusively on the presence of at least one EU/EEA investigational site. The United Kingdom (UK) was not considered part of the EU/EEA at the data cut-off. However, for eligible multinational trials, all participating countries listed in the databases were recorded to show broader geographic scope, including the UK and other countries outside the EU/EEA.

For each eligible study, the following variables were extracted: trial identification number; year of trial initiation within the EU/EEA; study phase; study status at the time of data extraction; study design; participating countries; demographic eligibility criteria; IMP orphan designation status.

For each identified IMP, the mechanism of action was determined and verified using publicly available regulatory information, clinical trial registry data, and, when necessary, peer-reviewed literature or official sponsor information. Based on the primary mechanism of action relevant to DMD, each IMP was assigned to a single therapeutic group: (1) dystrophin restoration or replacement; (2) inflammation, fibrosis, immune and epigenetic regulation; (3) muscle protection and regeneration; or (4) cardiopulmonary function.

Trial status was assessed at the time of data extraction and was standardised between the two registries. Trials reported as “Completed” in EudraCT or “Ended” in CTIS were classified as completed. Trials reported as “prematurely terminated” or “suspended”, or otherwise terminated before their planned completion, were classified as terminated. Trials reported as “Ongoing, recruiting” or “Ongoing, recruitment ended” were classified as ongoing. CTIS records with an “Authorised” status and no reported trial initiation were classified as authorised. Moreover, trials that were under an active temporary halt in the EU/EEA at the date cut-off were classified as temporarily halted. If a previously halted trial had restarted, it was classified according to its status at the time of data extraction.

All variables for the eligible studies were extracted manually. Due to the transitional period between registries, potential duplicate records were identified. Duplicate entries were carefully reviewed and cross-checked based on the study identifiers, sponsor information, and trial characteristics. ClinicalTrials.gov was additionally consulted to verify study identifiers and resolve duplications because of the transition from EudraCT to CTIS. All duplicates were merged to ensure that each trial was included only once in the final analysis. Orphan designation was verified and updated when needed using the EMA database of orphan medicinal products to reflect the up-to-date status of the IMP. For multinational trials, the year of initiation was defined as the year in which the study was first authorised in at least one EU/EEA country. Consequently, the EU/EEA initiation year may differ from the global initiation year of the trial.

The registry search, study selection, data extraction, and initial analysis were performed by one author (P.T.). The second author subsequently verified the eligibility decisions and extracted data (R.S.). Data management and statistical analyses were performed using Microsoft Excel (version 16.109, Microsoft Corporation, Redmond, WA, USA).

## 3. Results

The registry search identified a total of 95 records across EudraCT and CTIS. During manual deduplication, 15 duplicate records corresponding to transitional trials registered in both EudraCT and CTIS were identified and merged, resulting in 80 unique trials assessed for eligibility. After applying the inclusion criteria, 62 trials were included in the final analysis, while 18 were excluded. Complete characteristics of the included trials are summarised in Table S1. The study selection process is presented in Figure 1.

Of the 18 excluded records, 15 were DMD-specific trials conducted outside of the EU/EEA and registered in EudraCT in connection with paediatric regulatory requirements. Four were linked to an agreed PIP only, nine were associated with both PIP and Article 46, and two were submitted under Article 46 only. Another two trials had the UK as the only European study country. Both were registered while the UK was still an EU Member State. The remaining record was excluded because the indication for inclusion in the study was not limited to DMD. The distribution of excluded trials is presented in Figure 2, while detailed information on the individual excluded trials is provided in Table S2.

### 3.1. Operational Status at the Time of Data Extraction

Among the 62 included trials, 30.6% (*n* = 19) had been completed or ended at the time of the analysis, while 27.4% (*n* = 17) were ongoing. Premature termination was observed in 30.6% (*n* = 19) of studies, whereas 8.1% (*n* = 5) had been authorised but recruitment had not yet started. Two studies (3.2%) were temporarily halted within the EU/EEA because of safety-related concerns. These data are shown in Figure 3.

### 3.2. Distribution by Study Phase

Trial phase distribution is presented in Figure 4. Late-phase (Phase III) studies represented the largest proportion of included trials, accounting for 37.1% (*n* = 23), followed by Phase II studies at 30.6% (*n* = 19). Combined-phase studies (Phase I/II, Phase II/III and Phase I/II/III) comprised 21.0% (*n* = 13), whereas early-phase (Phase I) trials represented 9.7% (*n* = 6). Only one Phase IV study was identified during the study period.

Premature termination was observed in 2 of 6 Phase I trials (33.3%), 7 of 19 Phase II trials (36.8%), and 8 of 23 Phase III trials (34.8%), while 2 of 13 combined-phase studies (15.4%) were terminated. The single-Phase IV study was not terminated.

### 3.3. Study Design

The design characteristics of the included studies are summarised in Figure 5. Open-label studies represented half of all included trials (51.6%, *n* = 32). Placebo-controlled trials accounted for 30.6% (*n* = 19), while 17.7% (*n* = 11) included a two-part design of placebo-controlled and open-label study periods.

### 3.4. Temporal Trends in Clinical Trial Initiation

The annual distribution of clinical trial initiation in the EU/EEA is shown in Figure 6. Of the 62 included trials, 55 were initiated between 2016 and June 2026, two had started before 2016, and five had been authorised, but recruitment had not yet started. Clinical trial activity was observed throughout the entire study period, although the number of newly initiated studies varied between years. The highest number of initiated studies was recorded in 2024 (*n* = 10). Only one trial was initiated in 2022 and during the first six months of 2026. Overall, no consistent increasing or decreasing trend in trial initiation was found over the study period.

### 3.5. Geographic Distribution

Clinical trial activity was unevenly distributed across the EU/EEA (Figure 7, Table 1). Italy participated in the highest number of included trials (*n* = 43), followed by Spain and Belgium (*n* = 35 and 34, respectively), France (*n* = 25), the Netherlands (*n* = 23), and Germany (*n* = 20). In contrast, several EU/EEA countries were involved in only a small number of studies, including Slovenia and Romania (*n* = 1 each), while no DMD trials were identified in Croatia, Slovakia, Finland, Portugal, Estonia, Latvia, Lithuania, Cyprus, Malta, Iceland and Liechtenstein during the period analysed.

The included trials were frequently multinational. Among the countries outside of the EU/EEA, the United Kingdom was represented most frequently (*n* = 48), followed by the United States (*n* = 33), Canada (*n* = 29), Australia (*n* = 23), and Israel (*n* = 15). These numbers represent participation within the included EU/EEA-linked trial cohort and should not be interpreted as estimates of DMD clinical trial activity in these countries.

### 3.6. Disease Stage of Targeted Patient Population

The disease stage was categorised based on the protocol-specific inclusion criteria as ambulatory, non-ambulatory, mixed (both ambulatory and non-ambulatory), or not specified.

The disease stage of the study population varied across included clinical trials (Figure 8). Most studies included exclusively ambulatory patients (*n* = 26), while a smaller proportion included both ambulatory and non-ambulatory (*n* = 12). Trials enrolling non-ambulatory patients only were less frequent (*n* = 5). In 17 studies, disease stage was not explicitly specified in the publicly available inclusion/exclusion criteria and was therefore classified as “not specified”. Two Phase I studies were excluded from the analysis of this variable because the intended patient population was healthy subjects.

### 3.7. Therapeutic Approaches and IMPs

A total of 36 unique IMPs were identified across the 62 included trials. Of these, 35 were assigned to a single primary category based on their mechanism of action (Figure 9). Complete information on the identified IMPs is provided in Table S3. Imlifidase was counted among the 36 identified IMPs but was excluded from this classification because it is used exclusively as a pre-treatment before adeno-associated virus (AAV)-mediated gene therapy rather than as DMD-directed therapy.

Dystrophin-restoring or -replacing therapies accounted for 20 IMPs (57.1%), comprising 13 exon-skipping agents, four AAV-mediated gene therapies, two cell-based therapies, and one premature termination codon read-through agent. Seven IMPs (20.0%) were classified as therapies targeting inflammation, fibrosis, immune pathways, or epigenetic regulation. Five IMPs (14.3%) aimed to enhance muscle protection and regeneration, while three (8.6%) targeted cardiopulmonary function.

The number of clinical trials varied across IMPs. Vamorolone was evaluated in seven trials and was the only IMP investigated in a Phase IV study. Delandistrogene moxeparvovec was evaluated in five trials, followed by givinostat, which was investigated in four. The IMPs evaluated in at least three clinical trials are summarised in Table 2.

The 19 prematurely terminated trials involved 16 distinct IMPs: avicursen, BMN 044, BIO101, casimersen, delandistrogene moxeparvovec, domagrozumab, edasalonexent, eteplirsen, golodirsen, idebenone, metoprolol succinate, MNK-1411, pamrevlumab, suvodirsen, tamoxifen, and vesleteplirsen. Notably, all trials evaluating pamrevlumab, idebenone, and domagrozumab were prematurely terminated, alongside two of the three trials investigating suvodirsen.

When premature termination was assessed according to the therapeutic approach of the IMPs, differences were observed between the groups. The proportion of terminated trials was higher among studies targeting muscle protection and regeneration (4 of 6 trials) and cardiopulmonary function (3 of 4), whereas lower proportions were observed among trials investigating dystrophin restoration or replacement (7 of 34) and therapies targeting inflammation, fibrosis, immune pathways, or epigenetic regulation (5 of 18).

### 3.8. Orphan Designation of IMPs

Across the 62 included trials, a total of 36 unique IMPs were evaluated. Among these, 15 (41.7%) held an active EU orphan designation, whereas 16 (44.4%) did not. Orphan designations had been withdrawn or revoked for five IMPs (13.9%): fordadistrogene movaparvovec, pamrevlumab, edasalonexent, domagrozumab, and ataluren. Three of these five agents were associated with prematurely terminated trials in the dataset. The complete orphan designation status for all IMPs is presented in Figure 10.

## 4. Discussion

Across the 62 eligible trials, annual activity changed without showing a sustained upward or downward trend. Most studies were conducted in Phase II or III, but nearly one-third were terminated prematurely, and trial participation remained concentrated in a relatively small group of countries. Together, these findings describe the progress of drug development in DMD but also highlight the continuing scientific, operational, and geographic challenges. The combined analysis of EudraCT and CTIS also captures the transition between the previous and current regulatory frameworks in Europe.

### 4.1. Clinical Trial Activity

Although the number of newly initiated clinical trials varied between years, no consistent increase or decrease was observed during the study period. Clinical trials in rare diseases are associated with small, geographically dispersed patient populations and are influenced by their natural progression. These factors may restrict the number of studies that can be conducted simultaneously, prolong patient recruitment, prevent recruitment targets from being reached, and increase the operational complexity of multinational trials [16,17]. These challenges are also relevant to DMD trials, particularly given the difficulties associated with developing novel therapeutic approaches. For example, AAV-mediated gene therapies involve demanding manufacturing and uncertain durability of treatment effects [18]. Therefore, the absence of a clear increase in trial numbers should not necessarily be interpreted as stagnation in development. The relatively stable number of trials observed over the last decade may reflect the practical limitations of conducting trials in rare paediatric diseases.

### 4.2. Clinical Development

The high number of Phase II and Phase III studies in the present analysis suggests that a considerable proportion of therapies for DMD have progressed beyond early safety evaluation. This finding is consistent with the progress in DMD drug development over the past decade, during which several therapeutic approaches have ultimately led to marketing approval [19]. The relatively high proportion of combined-phase studies reflects the use of more flexible trial designs, allowing multiple stages of clinical development to be integrated within a single protocol and making more efficient use of the limited DMD patient population [20]. Such trials are particularly relevant in rare diseases, where patient recruitment is a challenge. By combining multiple stages within a single trial, they may shorten development timelines while reducing the burden associated with conducting multiple studies [21]. Another approach in the included studies was the two-part study design, usually consisting of a placebo-controlled period followed by open-label treatment. This design preserves a placebo-controlled comparison for efficacy assessment while allowing participants to subsequently receive the IMP.

Product-level analysis revealed that clinical development was concentrated within a relatively small number of therapies. Vamorolone and givinostat were among the most frequently investigated IMPs and subsequently received EU marketing authorisation in 2023 and 2025, respectively [6,7]. In contrast, the five trials evaluating delandistrogene moxeparvovec had mixed outcomes in terms of trial status at the time of data extraction—ongoing, completed, terminated, and temporarily halted studies. Although the product progressed to Phase III, the EMA recommended refusal of its marketing authorisation in 2025 because it did not demonstrate substantial clinical benefit [22]. The only two temporarily halted trials in the dataset, ENVISION and ENVOL, evaluated delandistrogene moxeparvovec and were paused in Europe following reports of fatal liver injuries in non-ambulatory patients [23]. In the United States, where delandistrogene moxeparvovec is authorised, the Food and Drug Administration (FDA) restricted its indication strictly to ambulatory patients and added a boxed warning regarding serious liver injury, including fatal outcomes [24]. Taken together, these findings underscore that even after reaching and conducting multiple late-phase trials, there may still be important safety concerns. Progression to late-phase development therefore does not always lead to favourable regulatory outcomes.

Exon-skipping therapies provide another example of a therapeutic approach for which regulatory uncertainty remains. Eteplirsen, golodirsen, viltolarsen, and casimersen were all identified within the EU/EEA clinical trial dataset and progressed to advanced development stages. All four received accelerated approval in the United States, with confirmatory studies required [25]. In contrast, no exon-skipping therapy is currently authorised in the EU. The marketing authorisation for eteplirsen was refused because its efficacy had not been sufficiently demonstrated [26]. The contrast between four approved exon-skipping therapies in the United States and none in the EU highlights substantial differences in regulatory assessment and decision-making between the two regions. Notably, no public EMA assessment reports were identified for golodirsen, viltolarsen, or casimersen; thus, the underlying reasons for the absence of EU marketing applications for these products may reflect product-specific development, regulatory, or commercial strategies of the respective sponsor.

### 4.3. Disease Stage of Trial Populations

Among studies in which disease stage could be determined, ambulatory patients were the most frequently represented population, while trials enrolling exclusively non-ambulatory patients were less common. This may partly reflect differences in functional outcome assessment between ambulatory and non-ambulatory DMD populations, as well as the challenges associated with evaluating treatment effects in more advanced disease stages [27].

Clinical investigation may also extend to more advanced disease stages over time. This was observed within the present dataset: givinostat was initially evaluated in the Phase III EPIDYS study enrolling ambulatory patients, while the subsequent Phase III ULYSSES study evaluated its efficacy in non-ambulatory patients.

However, ambulatory status could not be established from publicly available eligibility criteria for 17 studies. This relatively high proportion limits the interpretation of trial participation according to disease stage and highlights an important limitation of registry-based analyses of clinical trials, where detailed characteristics of the intended study population may not always be publicly accessible.

### 4.4. Trial Discontinuation

One of the main findings of this study was the high number of prematurely terminated clinical trials. Although trial termination is not rare in drug development, the observed termination rate highlights the considerable scientific and operational challenges associated with developing new therapies for DMD. According to publicly available registry information, the most frequently reported reasons for trial discontinuation included sponsor decisions, lack of efficacy, and safety concerns. However, these reasons were not reported consistently enough to support a formal comparison. Premature discontinuation remains an important part of the clinical development process. Discontinued trials may provide valuable information about certain therapeutic approaches, allowing resources to be redirected towards more promising candidates. This is relevant for DMD, where the genetic cause of the disease and the diversity of disease-causing mutations make the development of therapies that are both effective and safe particularly difficult.

When premature termination was examined across study phases, no clear trend was observed, as discontinued trials were identified in both early- and late-phase development. This suggests that progression to later phases of clinical development does not necessarily eliminate the risk of trial discontinuation.

Differences were also observed between therapeutic groups, with a higher termination rate among studies targeting muscle protection and regeneration and cardiopulmonary function than among the other two groups. However, these differences should be interpreted cautiously, as several therapeutic categories included only a small number of studies, and no formal association between therapeutic approach and premature termination was drawn.

Withdrawn orphan designation partly overlapped with prematurely terminated trials. Pamrevlumab, edasalonexent, and domagrozumab had withdrawn orphan designations and were associated with trials that were terminated following lack of efficacy or failure to meet a primary endpoint. However, the two events should not be interpreted as directly dependent on each other, as withdrawal of an orphan designation and trial termination are subject to separate regulatory and developmental decisions. Fordadistrogene movaparvovec and ataluren followed a different trajectory—both had their orphan designation withdrawn even when the corresponding trials in the dataset were not terminated.

### 4.5. Geographic Distribution

A geographic imbalance in the distribution of DMD clinical trials across the EU/EEA was observed. The highest levels of trial activity were present in Italy, Spain, Belgium, France and Germany. Similar geographic patterns have been described in other analyses of rare disease clinical research, where research activity is often concentrated in countries with established specialist centres, research networks, experienced investigators, and patient registries that facilitate patient identification and recruitment [28]. In the present analysis, however, these factors were not directly assessed. Therefore, the lower participation of several Central and Eastern European countries should be interpreted as a descriptive finding rather than as evidence of lower clinical research capacity across the EU/EEA.

On a global scale, the large number of trials with sites outside the EU/EEA shows the international nature of clinical development. The UK was the most frequently represented country outside the EU/EEA, participating in 48 of the 62 trials. The frequent involvement of countries such as the United States, Canada and Australia may help identify eligible patients and support recruitment across wider geographic areas, which is particularly important in rare diseases because of the small patient populations. In DMD, participation in clinical trials may be further restricted by age, underlying mutation, and other protocol-specific eligibility criteria.

### 4.6. Regulatory Considerations

An important methodological finding of this study was the difference in the scope of the two European clinical trial registries used for data collection. During data extraction, several records registered in EudraCT were identified despite having no investigational sites in the EU/EEA. These trials were included in the EU Clinical Trials Register because they formed part of an agreed PIP or fell under Article 46 of Regulation (EC) No. 1901/2006. Consequently, these studies appeared in EudraCT despite being entirely conducted outside of the EU/EEA. In contrast, no trials conducted outside of the EU/EEA were identified in CTIS. Information on certain paediatric trials continues to be submitted through EudraCT [29]. This distinction is important for registry analyses, as the presence of a study in EudraCT does not necessarily mean that the trial was conducted in Europe.

### 4.7. Strengths and Limitations

The combined use of registries, together with manual verification and extraction of detailed trial characteristics, enabled a comprehensive assessment of clinical trial activity during the transitional period between the two European regulatory frameworks. In addition to trial-specific variables, the analysis included the 36 individual IMPs, their mechanism of action, orphan designation and regulatory status. Nevertheless, several limitations should be acknowledged. The analysis relied exclusively on publicly available registry data, which may contain incomplete or outdated information due to protocol amendments or delays in updating trial records. Furthermore, the country-level participation does not show the number of individual centres or the number of participants. The number and phase of clinical trials do not provide evidence of IMP efficacy or safety and therefore cannot be used on their own to identify the most promising therapies. Lastly, the study was descriptive in nature and did not evaluate trial outcomes, treatment efficacy, or patient-level data apart from ambulatory status derived from eligibility criteria.

## 5. Conclusions

Between January 2016 and June 2026, DMD clinical trial activity in EU/EEA did not show a consistent temporal trend. Phase II and III studies accounted for more than two-thirds of the included trials, showing late-stage activity without necessarily indicating successful therapeutic development. The investigated products represent diverse therapeutic approaches, although more than half focused on restoring or replacing dystrophin. In this period, two new therapies received EU marketing authorisation. At the same time, the premature termination of 19 studies and the experience with delandistrogene moxeparvovec illustrate the challenges that remain in translating promising therapeutic approaches into safe and effective treatments. Trial participation remained uneven, with activity concentrated mainly in Western Europe, while several Central or Eastern European EU countries had limited or no trial participation. The large number of trials involving sites outside of the EU/EEA demonstrates the role of multinational trials in recruiting small and genetically defined populations. Ultimately, a better understanding of the EU/EEA clinical trial landscape may help identify opportunities to improve patient access to innovative therapies and strengthen future clinical research in DMD.

## Figures and Tables

**Figure 1:**
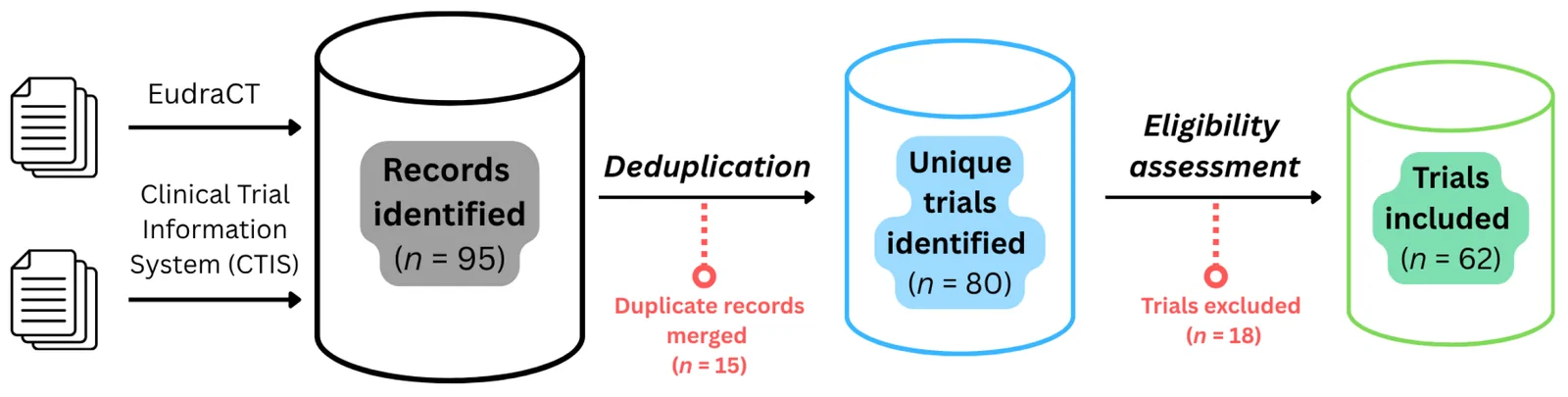
Flow diagram of clinical trial selection for the analysis (Created with https://www.Canva.com).

**Figure 2:**
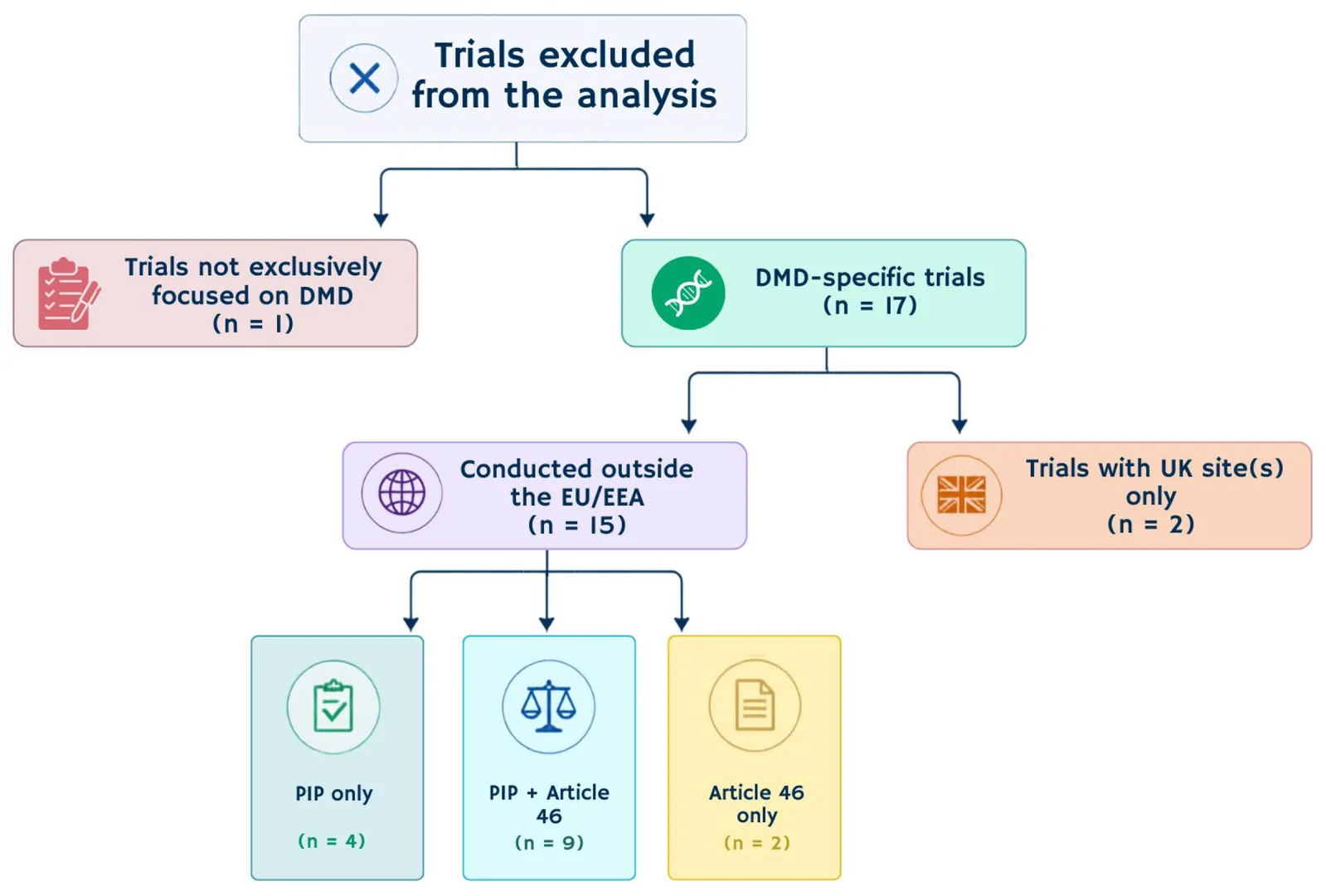
Classification of clinical trial records excluded from the primary analysis (*n* = 18). (Created with https://www.Canva.com).

**Figure 3:**
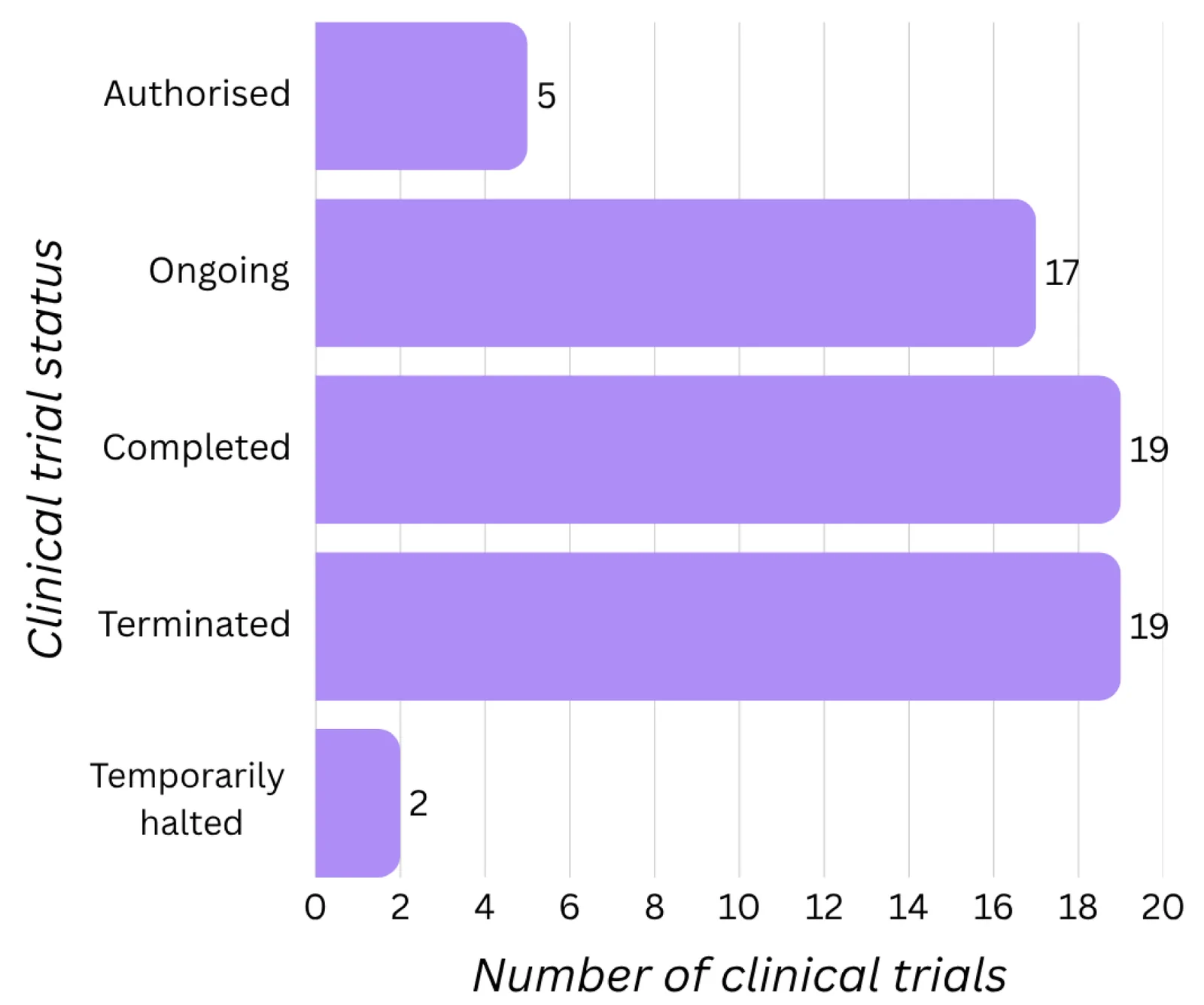
Status of the included trials in the analysis. Trials were classified according to their status in the CTIS and EudraCT databases at the time of data extraction. (Created with https://www.Canva.com).

**Figure 4:**
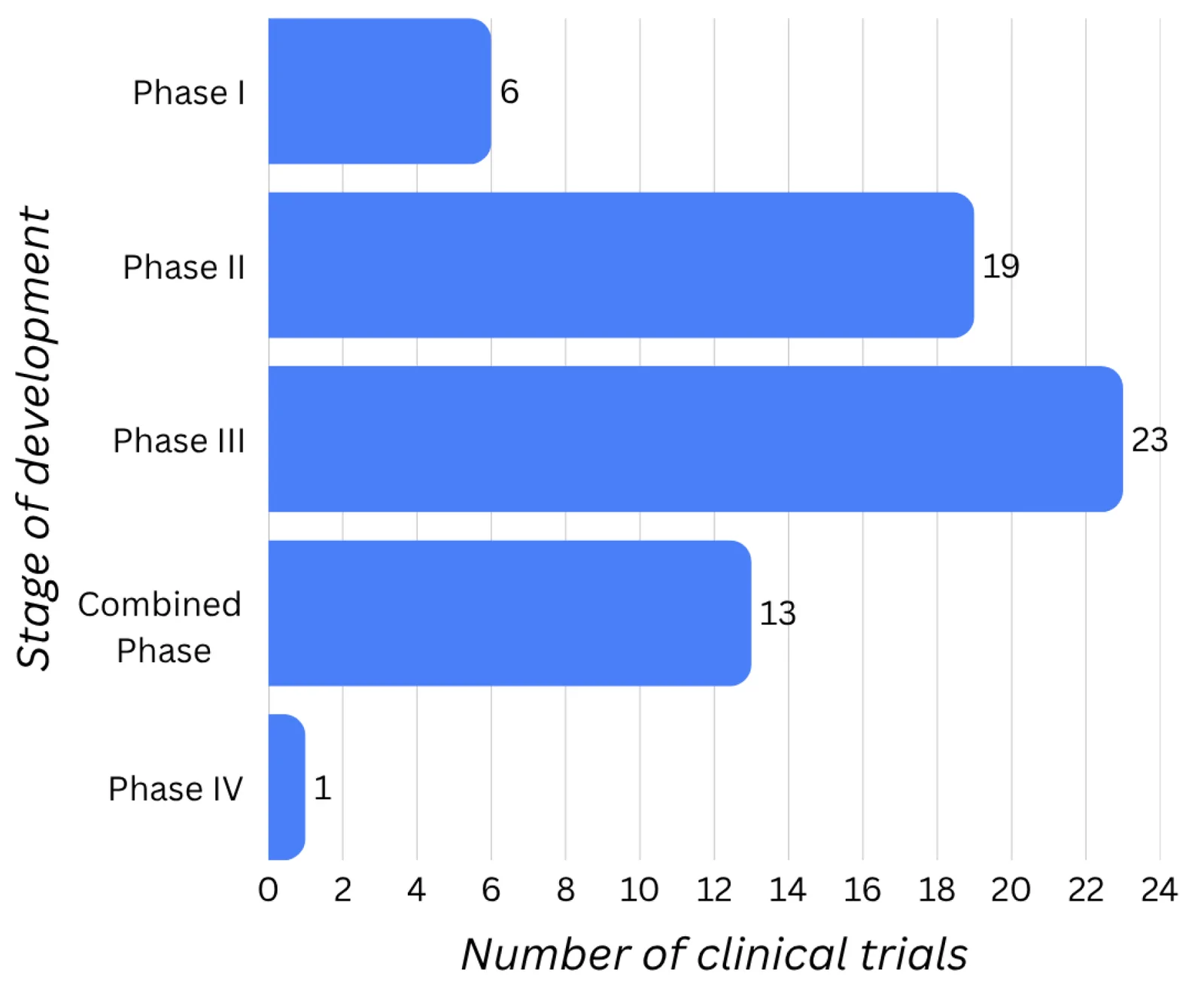
Distribution of the included clinical trials according to their study phase. (Created with https://www.Canva.com).

**Figure 5:**
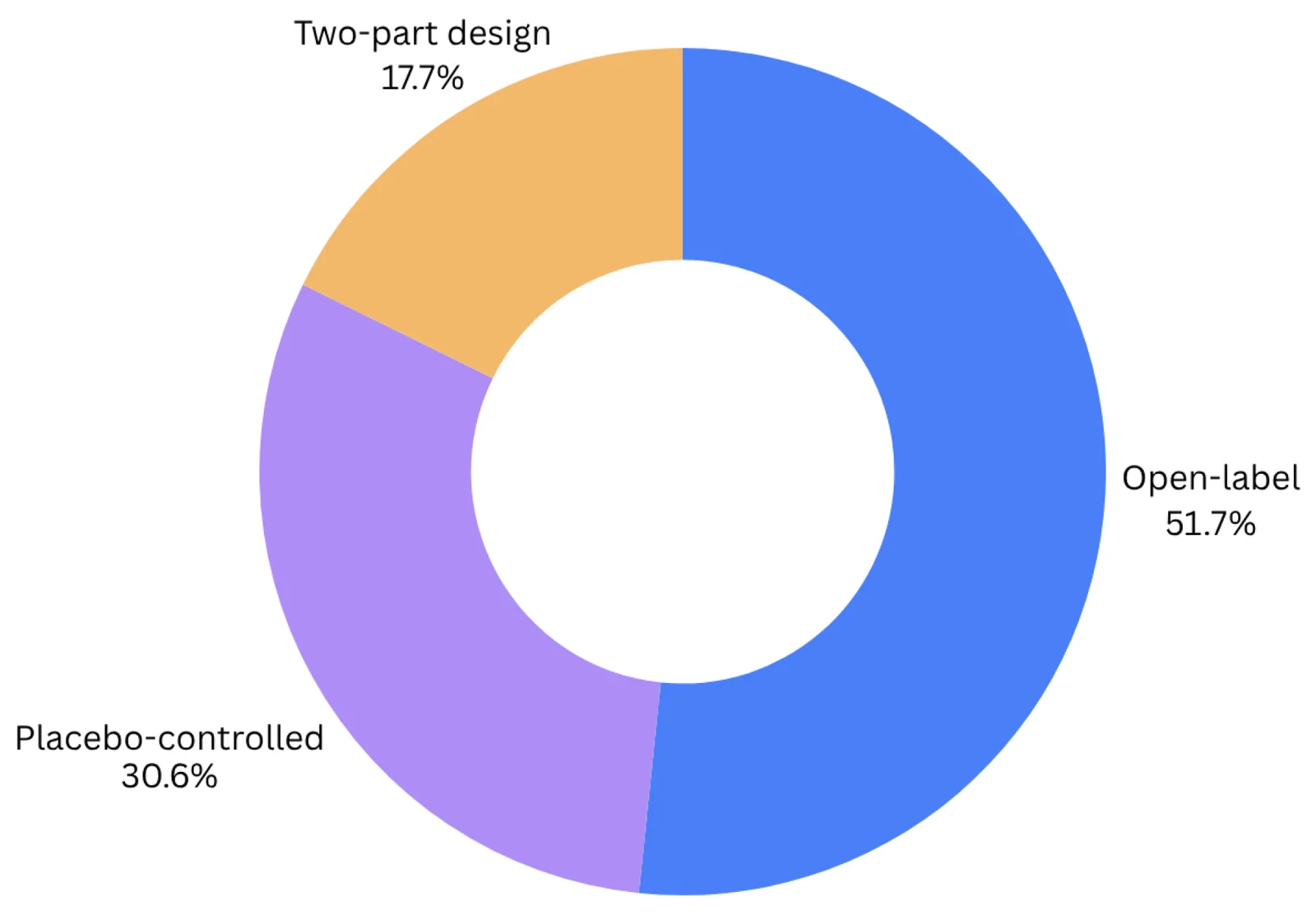
Distribution of the included clinical trials according to their study design. The “two-part” study design category includes studies that transition from a placebo-controlled period to open-label IMP administration within the same protocol. Open-label extension studies conducted under a different protocol are counted as separate clinical trials. (Created with https://www.Canva.com).

**Figure 6:**
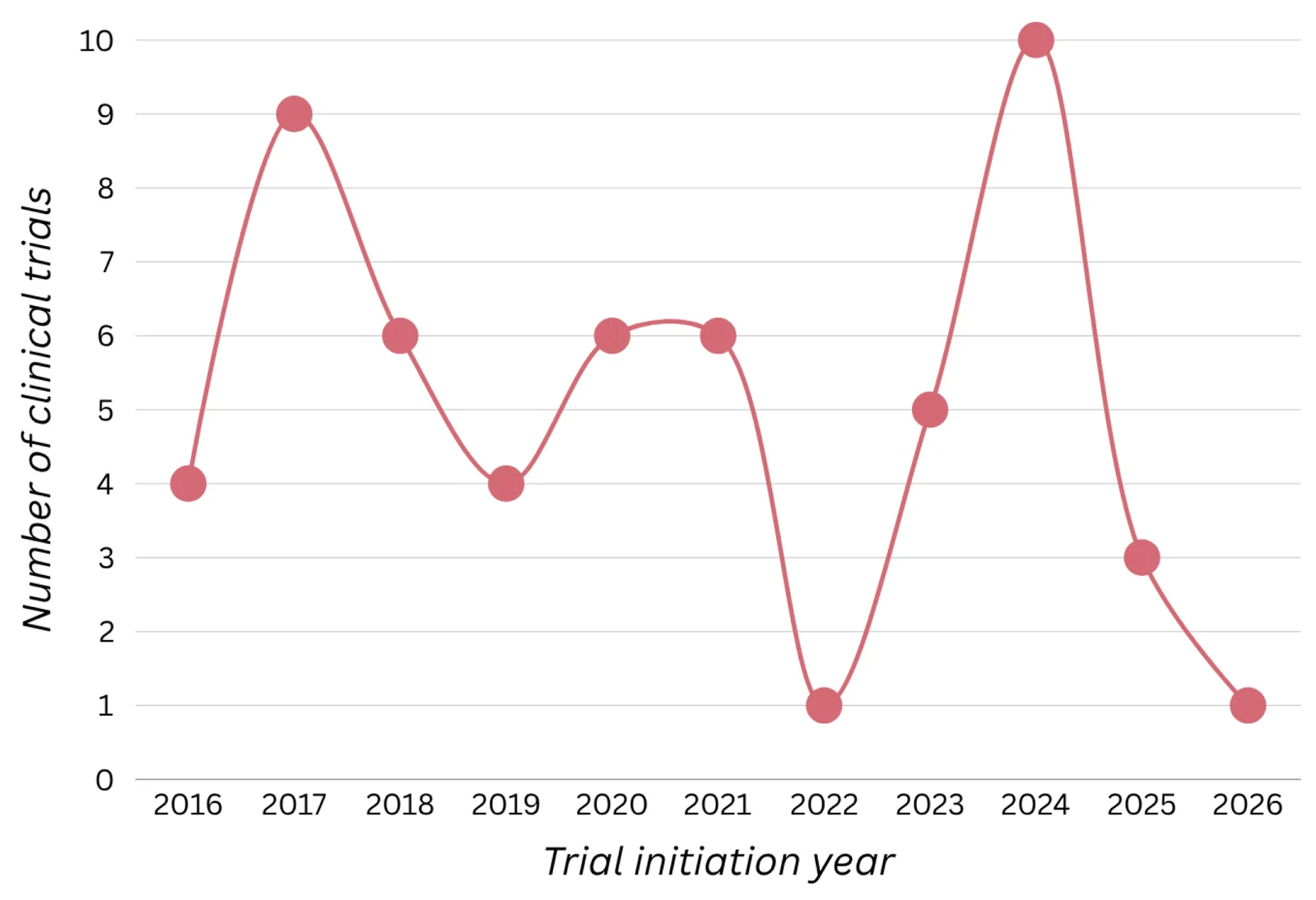
Annual initiation of DMD clinical trials in the EU/EEA from January 2016 to June 2026. The year shown corresponds to the first authorisation of the trial within the EU/EEA. (Created with https://www.Canva.com).

**Figure 7:**
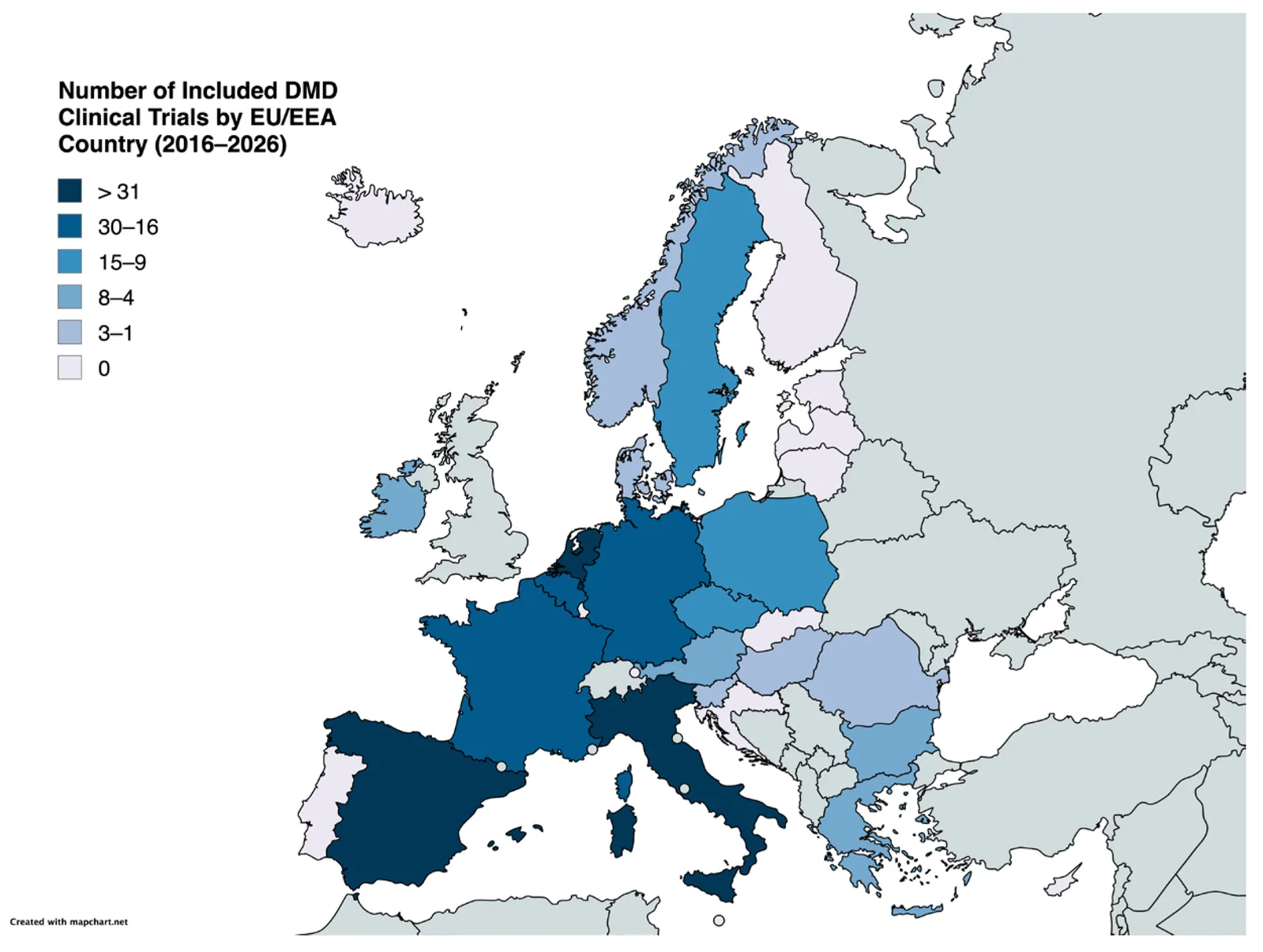
Geographic distribution of the included DMD clinical trials across the EU/EEA countries between January 2016 and June 2026. Countries outside the EU/EEA at the data cut-off are shown in grey. (Created with https://www.mapchart.net).

**Figure 8:**
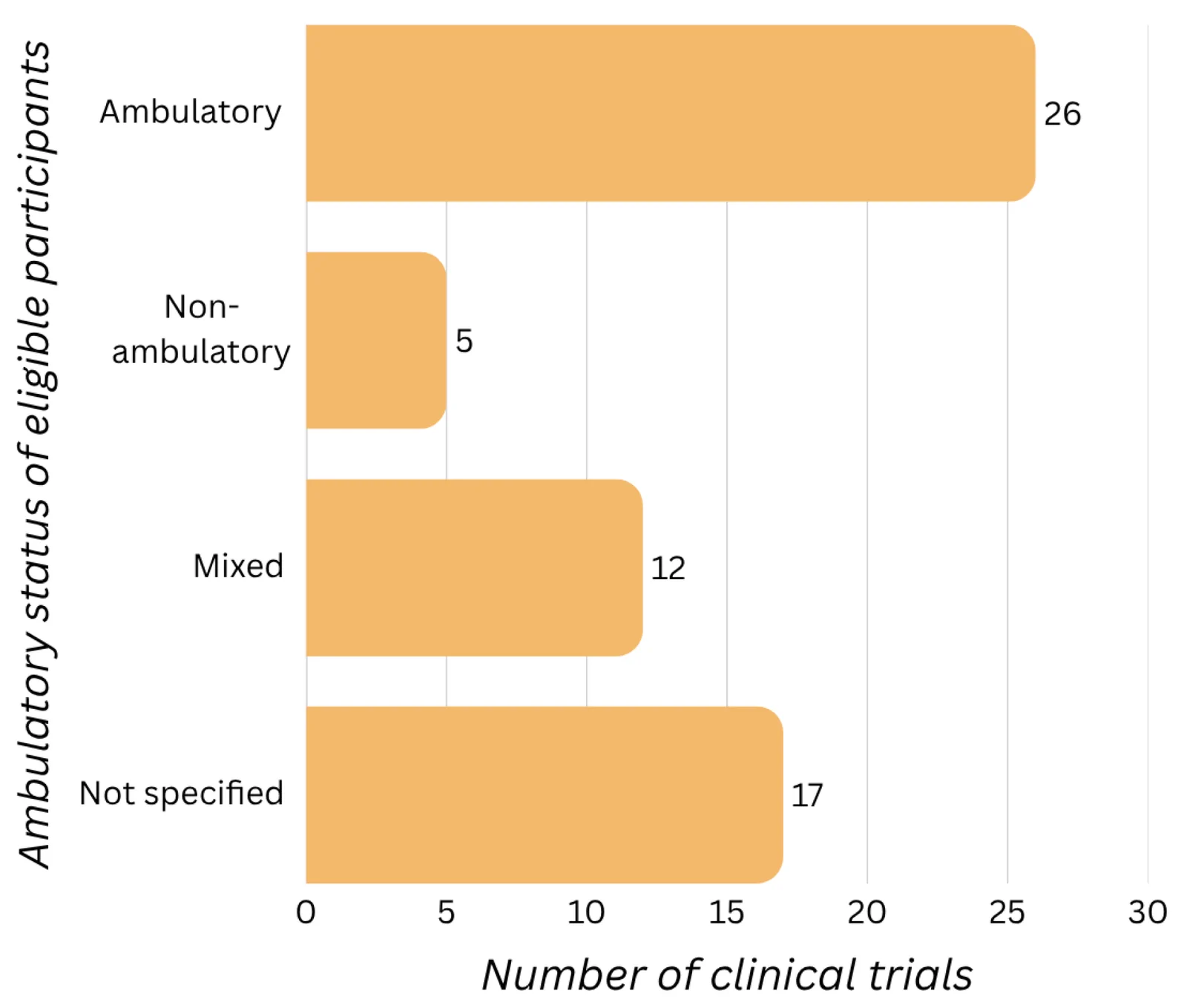
Distribution of included clinical trials according to the disease stage of eligible participants. Classification was based on the inclusion criteria specified in the clinical trial protocols of relevant studies. (Created with https://www.Canva.com).

**Figure 9:**
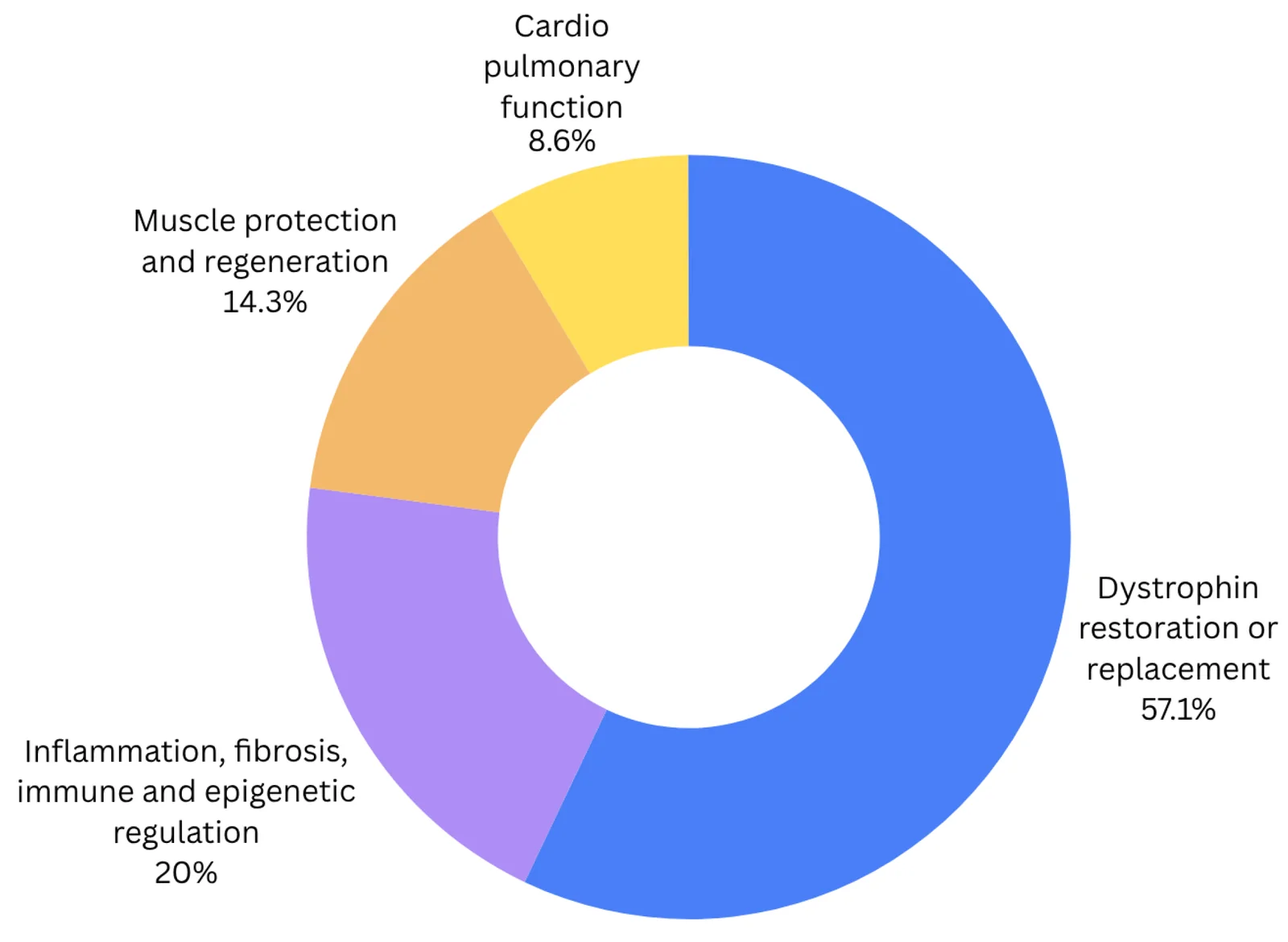
Distribution of the 35 classified IMPs for DMD according to their primary therapeutic approach. One IMP identified as a pre-treatment for AAV-mediated gene therapy was excluded from this classification. (Created with https://www.Canva.com).

**Figure 10:**
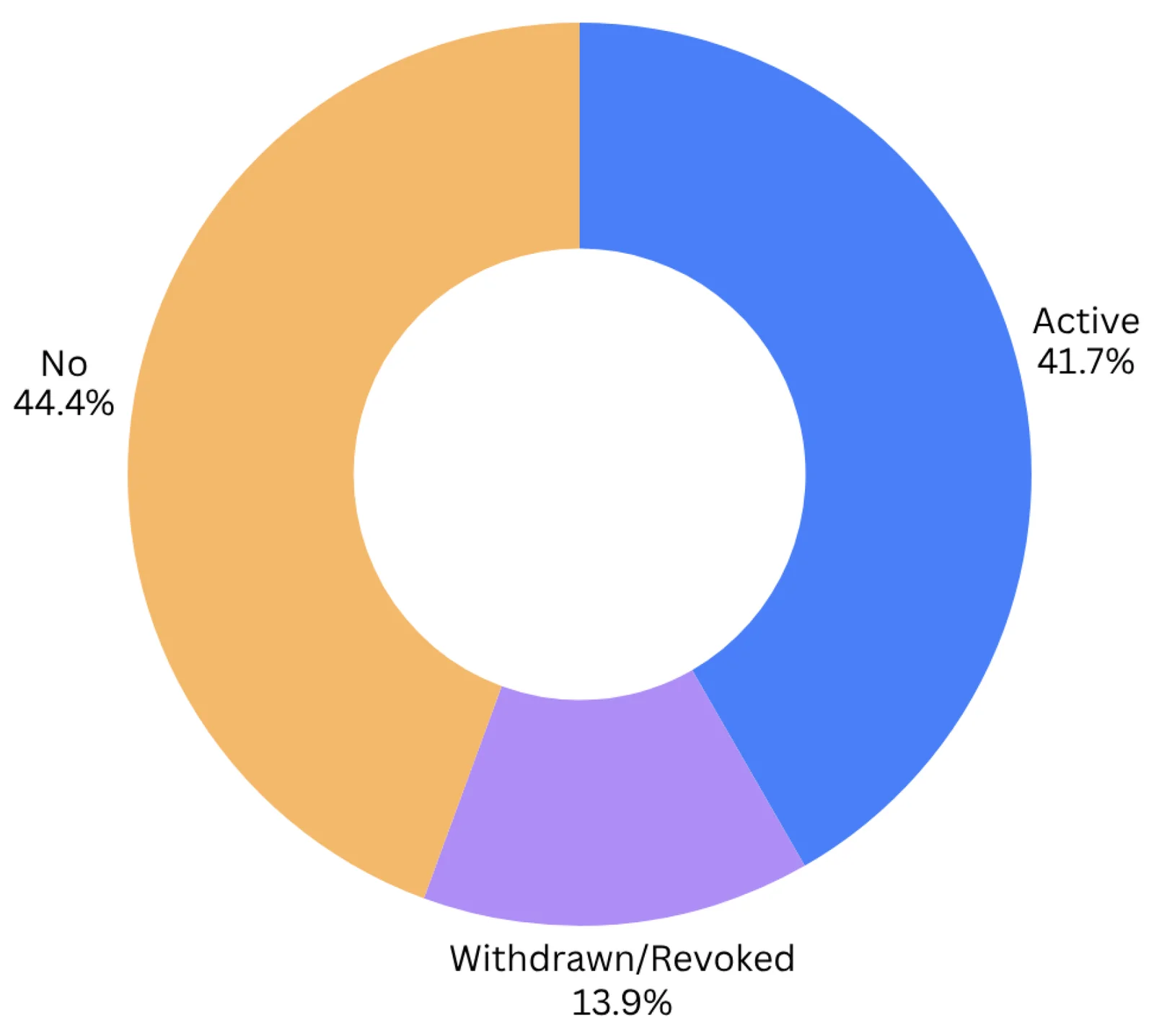
Orphan designation status in the EU of IMPs for which a clinical trial was conducted in the study period. The status was assessed in June 2026. (Created with https://www.Canva.com).

**Table 1:** The ten EU/EEA countries with the highest number of authorised trials for DMD between January 2016 and June 2026. Percentages were calculated using the 62 trials. As multinational trials were conducted in more than one country, percentages do not sum to 100%.

EU/EEA Country	Number of Trials (*n*)	Percentage (%)
Italy	43	69.4%
Spain	35	56.5%
Belgium	34	54.8%
France	25	40.3%
Netherlands	23	37.1%
Germany	20	32.3%
Sweden	13	21.0%
Poland	11	17.7%
Czechia	9	14.5%
Bulgaria	8	12.9%

**Table 2:** IMPs represented in at least three included trials.

IMP	Therapeutic Approach	Number of Clinical Trials (*n*)
Vamorolone	Corticosteroid	7
Delandistrogene	AAV-mediated gene therapy	5
Givinostat	Histone deacetylase inhibitor	4
Eteplirsen	Exon 51 skipping	3
Golodirsen	Exon 53 skipping	3
Suvodirsen	Exon 51 skipping	3
Viltolarsen	Exon 53 skipping	3

## Data Availability

The datasets analyzed during the current study are available from the corresponding author upon reasonable request.

## Supplementary Materials

The following supporting information can be downloaded at: https://www.mdpi.com/article/10.3390/medicina62101921/s1, Table S1: Characteristics of the included clinical trials in the primary analysis; Table S2: Clinical trials excluded from the primary analysis and reasons for exclusion; Table S3: Investigational medicinal products identified in the included clinical trials and their main characteristics.

## Abbreviations

The following abbreviations are used in this manuscript:

AAV	Adeno-associated virus
CTIS	Clinical Trials Information System
DMD	Duchenne Muscular Dystrophy
EMA	European Medicines Agency
EU	European Union
EEA	European Economic Area
EudraCT	European Union Drug Regulating Authorities Clinical Trial Database
FDA	Food and Drug Administration
IMP	Investigational Medicinal Product
PIP	Paediatric Investigation Plan
UK	United Kingdom
