# Supplementary material for: Clinical Trial Landscape of Duchenne Muscular Dystrophy in Europe: A 10-Year Retrospective Analysis
Source: Medicina (Kaunas). 2026 Oct 5;62(10):1921. doi: 10.3390/medicina62101921 (PMC13706884; doi:10.3390/medicina62101921)
Supplement: Supplementary file 1 [file medicina-62-01921-s001.zip › medicina-4515655-supplementary.pdf]

Table S1. Characteristics of the included clinical trials in the primary analysis.

Protocol Number	EudraCT Number	CTIS Number	NCT Number	Trial acronym	Sponsor	IMP(s)	Phase	Trial Status	EU/EEA Start year	EU/EEA End year	Trial Design	Ambulatory Status of eligible participants	EU/EEA participating countries	Other participating countries
DT-DEC01-DMD-CT-1/2	2022-003126-42	2024-519004-27-00	N/A	DEC for DMD	Dystrogen Therapeutics Technology Polska Spółka z Ograniczoną Odpowiedzialnością	DT-DEC01	I/II	Authorised	N/A	N/A	Open label	Mixed	Poland	None
ENTR-601-DMD-202	N/A	2025-525124-10-00	N/A	ELEVATE-LTE	Entrada Therapeutics	ENTR-601-44, ENTR-601-45	II	Authorised	N/A	N/A	Open label	Mixed	Belgium, Italy, Netherlands, Spain	United Kingdom
SGT-003-301	N/A	2025-522949-22-00	NCT07160634	IMPACT DUCHENNE	Solid Biosciences	SGT-003	III	Authorised	N/A	N/A	Two-part design	Ambulatory	Belgium, France, Italy, Netherlands, Spain	Australia, Canada, United States
AOC 1044-CS3	N/A	2025-523087-20-00	NCT07587242	SAFARI44	Avidity Biosciences	AOC 1044	III	Authorised	N/A	N/A	Two-part design	Ambulatory	Belgium, France, Italy, Germany, Netherlands, Poland, Spain	Australia, Canada, United Kingdom
351-202	N/A	2024-511656-41-00	NCT07573631	N/A	Biomarin Pharmaceutical	BMN 351	II	Authorised	NA	N/A	Open label	Ambulatory	Italy, Netherlands, Spain	Turkey, United Kingdom
SAT-3247-CL-201	N/A	2025-522522-13-01	NCT07287189	BASECAMP	Satellos Bioscience	SAT-3247 OXALATE	II	Ongoing	2026	N/A	Two-part design	Ambulatory	Belgium, Poland, Spain	Australia, Canada, Serbia, United

														Kingdom, United States
ENTR-601-45-201	N/A	2024-517499-39-00	NCT07038824	ELEVATE-45	Entrada Therapeutics	ENTR-601-45	I/II	Ongoing	2025	N/A	Two-part design	Mixed	Belgium, Italy, Netherlands, Spain	United Kingdom
ENTR-601-44-201	N/A	2024-517584-23-00	NCT07037862	ELEVATE-44	Entrada Therapeutics	ENTR-601-44	I/II	Ongoing	2025	N/A	Two-part design	Mixed	Belgium, Italy, Spain	United Kingdom
SGT-003-101	N/A	2024-514501-57-00	NCT06138639	INSPIRE DUCHENNE	Solid Biosciences	SGT-003	I/II	Ongoing	2025	N/A	Open label	Mixed	Italy	Canada, United Kingdom, United States
SRP-9001-303	2020-002372-13	2024-512626-28-00	NCT05881408	ENVISION	Sarepta Therapeutics	Delandistrogene moxeparvovec-rokl	III	Temporarily halted	2024	N/A	Placebo-controlled	Mixed	Belgium, France, Germany, Italy, Spain, Sweden	Australia, Canada, Hong Kong, Israel, Japan, South Korea, Taiwan, United Kingdom, United States
SNT-IV-VAM-011	N/A	2024-512828-12-00	NCT06713135	The GUARDIAN Study	Santhera Pharmaceuticals	Vamorolone	IV	Ongoing	2024	N/A	Open label	Mixed	Belgium, Czechia, Greece, Ireland, Netherlands, Spain	Australia, Israel, New Zealand, United Kingdom
BN45398	N/A	2024-512383-65-00	NCT06450639	SHIELD DMD	F. Hoffmann-La Roche AG	Satralizumab	II	Ongoing	2024	N/A	Open label	Mixed	Poland, Spain, Italy, Denmark	Japan, Ukraine, United States
DSC/14/2357/50	N/A	2023-503521-19-00	NCT05933057	ULYSSES	Italfarmaco	Givinostat	III	Ongoing	2024	N/A	Placebo-controlled	Non-ambulatory	Belgium, Czechia, France,	Canada, United Kingdom

													Germany, Italy, Netherlands, Poland, Sweden	
351-201	N/A	2023-506737-30-00	NCT06280209	N/A	Biomarin Pharmaceutical	BMN 351	I/II	Ongoing	2024	N/A	Open label	Ambulatory	Italy, Netherlands, Spain	Turkey, United Kingdom
DSC/14/2357/52	N/A	2024-511823-32-00	NCT06769633	N/A	Italfarmaco	Givinostat	II	Ongoing	2024	N/A	Open label	Not specified	Belgium, Italy, Netherlands	United Kingdom
DMD06-MAB	2023-000148-47	2024-514860-14-00	N/A	N/A	Ospedale San Raffaele S.r.l., University Of Manchester	DMD06-MAB	I/II	Ongoing	2024	N/A	Open label	Non-ambulatory	Italy	None
SNT-I-VAM-025	N/A	2024-513845-36-00	NCT06689527	N/A	Santhera Pharmaceuticals	Vamorolone (and midazolam)	I	Completed	2024	2024	Open label	N/A	Germany	None
SNT-I-VAM-026	N/A	2024-512101-60-00	NCT06649409	LIONHEART	Santhera Pharmaceuticals	Vamorolone (and fludrocortisone and eplerenone)	I	Completed	2024	2024	Open label	N/A	Germany	None
SRP-9001-305	N/A	2023-505043-39-00	NCT05967351	EXPEDITION	Sarepta Therapeutics	Delandistrogene moxeparvovec-rokl	III	Ongoing	2024	N/A	Open Label	Not specified	Belgium, Germany, Italy, Spain	Australia, Hong Kong, Israel, Japan, Taiwan, United Kingdom, United States
BN43881	2022-000691-19	2023-509901-57-00	NCT06128564	ENVOL	F. Hoffmann-La Roche AG	Delandistrogene moxeparvovec-rokl	II	Temporarily halted	2023	N/A	Open label	Not specified	Belgium, France, Germany, Italy, Spain	United Kingdom

1102-DMD-Pre-CT03	2022-003065-38	2024-512265-13-00	NCT05938023	N/A	Antisense Therapeutics	Avicursen (ATL1102)	II	Terminated	2023	2025	Two-part design	Non-ambulatory	Bulgaria	Australia, Serbia, Turkey, United Kingdom
AVANCE 1-1/2a	N/A	2022-500703-49-01	NCT05753462	ADVANCE 1	Sqy Therapeutics	SQY51	I/II	Ongoing	2023	N/A	Open label	Mixed	France	None
SRP-9001-104	2022-003407-15	2024-512624-11-00	NCT06241950	N/A	Sarepta Therapeutics	Delandistrogene moxeparvovec-rokl, Imlifidase	I	Terminated	2023	2025	Open label	Ambulatory	Spain	None
DYNE251-DMD-201	2021-005478-24	2023-510351-31-00	NCT05524883	DELIVER	Dyne Therapeutics	DYNE-251	I/II	Ongoing	2023	N/A	Placebo-controlled	Mixed	Belgium, Ireland, Italy, Spain	Australia, Canada, South Korea, United Kingdom, United States
NS-065/NCNP-01-302	2021-000122-10	2023-507146-91-00	NCT04768062	RACER53-X	Ns Pharma	Viltolarsen	III	Completed	2021	2025	Open label	Ambulatory	Czechia, Greece, Italy, Netherlands, Norway, Spain	Australia, Canada, Chile, China, Mexico, New Zealand, Russia, South Korea, Turkey, United Kingdom
FGCL-3019-094	2020-000699-39	N/A	NCT04632940	LELANTOS-2	FibroGen	Pamrevlumab	III	Terminated	2021	2023	Placebo-controlled	Ambulatory	Austria, Belgium, France, Italy, Netherlands, Spain	Australia, Canada, China, United Kingdom

SRP-9001-301	2019-003374-91	N/A	NCT05096221	EMBARK	Sarepta Therapeutics	Delandistrogene moxeparvovec-rokl	III	Completed	2022	2024	Placebo-controlled	Ambulatory	Belgium, Germany, Italy, Spain	Hong Kong, Japan, Taiwan, United Kingdom, United States
NS-065/NCNP-01-211	2020-003653-30	N/A	NCT04956289	Galactic53	Ns Pharma	Viltolarsen	II	Completed	2021	2023	Open label	Mixed	Italy, Spain	China, Russia, Turkey, United States
GNT-016-MDYF	2020-002093-27	2023-505187-11-00	N/A	N/A	Genethon	GNT0004	I/II/III	Ongoing	2021	NA	Two-part design	Ambulatory	Belgium, France, Spain	United Kingdom
NBK154/1/2020	2020-004901-29	2024-515057-19-00	NCT05066633	MeDMD	Medical University Of Gdansk	metoprolol succinate	III	Terminated	2021	2025	Placebo-controlled	Not specified	Poland	None
4658-402	2018-001762-42	2024-511492-15-00	NCT03992430	MIS51ON	Sarepta Therapeutics	Eteplirsen	III	Ongoing	2021	NA	Open label	Ambulatory	Czechia, Denmark, France, Germany, Greece, Hungary, Italy, Netherlands, Norway, Poland, Romania, Slovenia, Spain	India, Jordan, Mexico, New Zealand, Serbia, South Korea, Switzerland, Taiwan, Turkey, United Kingdom, United States
NS-065/NCNP-01-301	2019-002076-13	N/A	NCT04060199	RACER53	Ns Pharma	Viltolarsen	III	Completed	2020	2023	Placebo-controlled	Ambulatory	Greece, Italy, Netherlands, Norway, Spain	Australia, Canada, Chile, China, Hong Kong, Mexico, New Zealand, Russia, South Korea,

														Taiwan, Turkey, Ukraine, United Kingdom, United States
C3391003	2019-002921-31	2023-508510-42-00	NCT04281485	CIFFREO	Pfizer	Fordadistrogene Movaparvovec (PF-06939926)	III	Ongoing	2020	NA	Placebo-controlled	Ambulatory	Belgium, France, Germany, Italy, Spain	Australia, Canada, Israel, Japan, Russia, South Korea, Switzerland, Taiwan, United Kingdom, United States
FGCL-3019-093	2020-000698-26	N/A	NCT04371666	LELANTOS-1	FibroGen	Pamrevlumab	III	Terminated	2020	2023	Placebo-controlled	Non-ambulatory	Austria, Belgium, Czechia, France, Italy, Netherlands, Spain	Australia, Canada, China, Israel, Switzerland, United Kingdom, United States
BIO101_CL04	2019-004602-94	N/A	N/A	N/A	Biophytis	BIO101 (Sarconeos)	I/II/III	Terminated	2020	2020	Placebo-controlled	Non-ambulatory	Belgium	None
5051-201	2019-000601-77	2023-509935-23-00	NCT04004065	MOMENTUM	Sarepta Therapeutics	Vesleteplirsen (SRP-5051)	II	Terminated	2020	2024	Open label	Not specified	Belgium, Germany, Italy, Netherlands, Spain	Canada, United Kingdom, United States
WVE-DMDX51-003	2018-004009-22	N/A	NCT03907072	DYSTANCE 51	Wave Life Sciences	Suvodirsen (WVE-210201)	II/III	Terminated	2019	2020	Placebo-controlled	Ambulatory	Belgium, Czechia, France, Italy, Sweden	Canada, United Kingdom, United States

CAT-1004-302	2019-003563-22	N/A	NCT03917719	GalaxyDMD	Catabasis Pharmaceuticals	Edasalonexent	III	Terminated	2020	2020	Open label	Ambulatory	Germany, Sweden	Australia, Canada, United Kingdom, United States
4658-102-OLE	2019-000337-39	N/A	NCT03985878	N/A	Sarepta Therapeutics	Eteplirsen	II	Terminated	2019	2022	Open label	Not specified	Belgium, France, Italy	United Kingdom
4045-302	2017-004625-32	N/A	NCT03532542	N/A	Sarepta Therapeutics	Casimersen or Golodirsen	III	Terminated	2018	2023	Open label	Not specified	Belgium, Bulgaria, Czechia, France, Germany, Italy, Poland, Spain, Sweden	Canada, Israel, United Kingdom, United States
VBP15-004-A4	2017-002704-27	N/A	NCT03439670	VISION DMD	ReveraGen Biopharma	Vamorolone	II	Completed	2019	2021	Placebo-controlled	Ambulatory	Belgium, Czechia, Greece, Netherlands, Spain, Sweden	Australia, Canada, Israel, United Kingdom, United States
SNT-III-012-E	2017-004279-30	N/A	NCT03603288	SIDEROS-E	Santhera Pharmaceuticals	Idebenone	III	Terminated	2018	2020	Open label	Not specified	Austria, Belgium, France, Germany, Italy, Spain	Switzerland, United Kingdom, United States
MNK14112096	2017-004139-35	N/A	NCT03400852	BRAVE	Mallinckrodt ARD	MNK-1411	II	Terminated	2018	2020	Placebo-controlled	Not specified	Bulgaria, Italy, Spain	Israel, Mexico, Serbia, Turkey, United States
CAT-1004-301	2018-000464-29	N/A	NCT03703882	POLARIS DMD	Catabasis Pharmaceuticals	Edasalonexent	III	Completed	2019	2020	Placebo-controlled	Ambulatory	Germany, Ireland, Sweden	Australia, Canada, Israel, United

														Kingdom, United States
WVE-DMDX51-001	2017-002686-21	N/A	NCT03508947	N/A	Wave Life Sciences	Suvodirsen (WVE-210201)	I	Completed	2018	2019	Placebo-controlled	Mixed	Belgium, France, Italy, Netherlands	Canada, United Kingdom
TAMDMD	2017-004554-42	N/A	NCT03354039	TAMDMD	University of Basel Children's Hospital, Division of Neuropediatrics	Tamoxifen	III	Terminated	2018	2022	Two-part design	Ambulatory	France, Germany, Netherlands, Spain	Switzerland, United Kingdom
WVE-DMDX51-002	2018-000975-34	N/A	N/A	N/A	Wave Life Sciences	Suvodirsen (WVE-210201)	I	Terminated	2018	2020	Open label	Not specified	Belgium, France, Italy	Canada, United Kingdom, United States
PTC124-GD-041DMD	2017-001223-49	N/A	NCT03179631	N/A	PTC Therapeutics	Ataluren	III	Completed	2017	2023	Two-part design	Ambulatory	Bulgaria, Poland	Australia, Brazil, Canada, China, Hong Kong, India, Japan, Malaysia, Mexico, Puerto Rico, Russia, South Korea, Taiwan, Thailand, Turkey, United States
WN40227	2016-001654-18	N/A	NCT03039686	SPITFIRE	F. Hoffmann-La Roche AG	RO7239361	II/III	Completed	2017	2020	Placebo-controlled	Ambulatory	Belgium, France, Germany, Italy,	Argentina, Australia, Canada, Japan, United

												Netherlands, Sweden	Kingdom, United States
DSC/14/2357/48	2016-000401-36	N/A	NCT02851797	EPIDYS	Italfarmaco	Givinostat	III	Completed	2017	2022	Placebo-controlled	Ambulatory	Belgium, France, Germany, Italy, Netherlands, Spain	Canada, Israel, Serbia, United Kingdom, United States
DSC/14/2357/51	2017-000397-10	2023-504520-26-00	NCT03373968	N/A	Italfarmaco	givinostat	III	Ongoing	2017	NA	Open label	Not specified	Belgium, France, Germany, Italy, Netherlands, Spain	Canada, Israel, Serbia, United Kingdom, United States
VBP15-LTES	2017-003568-10	N/A	NCT03038399	N/A	ReveraGen Biopharma	Vamorolone	II	Completed	2017	2020	Open label	Not specified	Sweden	Australia, Canada, Israel, United Kingdom, United States
4658-102	2016-000951-29	N/A	NCT03218995	N/A	Sarepta Therapeutics	Eteplirsen	II	Completed	2017	2021	Open label	Not specified	Belgium, France, Italy	United Kingdom
4045-301	2015-002069-52	2024-514698-23-00	NCT02500381	ESSENCE	Sarepta Therapeutics	golodirsen (SRP-4045), casimersen (SRP-4053)	III	Completed	2017	2025	Two-part design	Not specified	Belgium, Bulgaria, Czechia, Denmark, Hungary, Ireland, Italy, Poland, Spain	Argentina, Australia, Mexico, Russia, Serbia, South Korea, United Kingdom
SNT-III-012	2016-000602-10	N/A	NCT02814019	SIDEROS	Santhera Pharmaceuticals	Idebenone	III	Terminated	2016	2020	Placebo-controlled	Not specified	Austria, Belgium, Bulgaria, France, Germany,	Israel, Switzerland, United Kingdom, United States

													Hungary, Ireland, Italy, Netherlands, Spain, Sweden	
VBP15-002	2016-004262-26	N/A	NCT02760264	N/A	ReveraGen Biopharma	Vamorolone	II	Completed	2017	2018	Open label	Ambulatory	Sweden	Australia, Canada, Israel, United Kingdom, United States
VBP-003	2016-004263-38	N/A	NCT02760277	N/A	ReveraGen Biopharma	Vamorolone	II	Completed	2017	2018	Open label	Not specified	Sweden	Australia, Canada, Israel, United Kingdom, United States
B5161004	2016-001615-21	N/A	NCT02907619	N/A	Pfizer	Domagrozumab (PF-06252616)	II	Terminated	2016	2018	Open label	Ambulatory	Bulgaria, Italy	Canada, Japan, United Kingdom, United States
EspeRare_RIM_001	2015-002530-50	N/A	NCT02710591	RIM4DMD	EspeRare Foundation	Rimeporide	I	Completed	2016	2018	Open label	Ambulatory	France, Italy, Spain	United Kingdom
BMN-044-201	2015-003681-87	N/A	NCT02958202	N/A	Biomarin Pharmaceutical	BMN 044	II	Terminated	2016	2016	Open label	Not specified	Belgium, Italy, Netherlands, Sweden	None
4053-101	2014-002008-25	N/A	NCT02310906	SKIP-NMD	Sarepta Therapeutics	SRP-4053	I/II	Completed	2014	2019	Two-part design	Ambulatory	France, Italy	United Kingdom, United States
B5161002	2014-002072-92	N/A	NCT02310763	N/A	Pfizer	Domagrozumab (PF-06252616)	II	Terminated	2014	2018	Placebo-controlled	Ambulatory	Bulgaria, Italy, Poland	Australia, Canada, Japan, United Kingdom, United States

Table S2. Clinical trials excluded from the primary analysis and reasons for exclusion.

Protocol Number	EudraCT Number	CTIS Number	NCT Number	Trial acronym	Sponsor	IMP(s)	Countries	Reason for exclusion
5051-102	2020-004803-15	N/A	NCT03675126	N/A	Sarepta Therapeutics	SRP-5051 (vesleteplirsen)	United States, Canada	Conducted entirely outside the EU/EEA; registered in relation to a PIP
FGCL-3019-079	2023-000321-80	N/A	NCT02606136	MISSON	FibroGen	Pamrevlumab	United States	Conducted entirely outside the EU/EEA; registered in relation to a PIP and Article 46
4658-301	2016-005002-19	N/A	NCT02255552	PROMOVI	Sarepta Therapeutics	Eteplirsen	United States	Conducted entirely outside the EU/EEA; registered in relation to a PIP
4658-204	2016-005024-28	N/A	NCT02286947	N/A	Sarepta Therapeutics	Eteplirsen	United States	Conducted entirely outside the EU/EEA; registered in relation to a PIP and Article 46
4658-us-202	2016-005001-39	N/A	NCT01540409	N/A	Sarepta Therapeutics	Eteplirsen	United States	Conducted entirely outside the EU/EEA; registered in relation to a PIP and Article 46
4658-203	2016-005023-92	N/A	NCT02420379	N/A	Sarepta Therapeutics	Eteplirsen	United States	Conducted entirely outside the EU/EEA; registered in relation to a PIP
VBP15-006	2025-000201-16	N/A	NCT05185622	N/A	Santhera Pharmaceuticals	Vamorolone	Canada	Conducted entirely outside the EU/EEA; registered in relation to a PIP and Article 46
SRP-9001-102	2021-000078-27	N/A	NCT03769116	N/A	Sarepta Therapeutics	Delandistrogene moxeparvovec-rokl	United States	Conducted entirely outside the EU/EEA; registered in relation to a PIP and Article 46

PTC124-GD-030-DMD	2016-001764-11	N/A	NCT02819557	N/A	PTC Therapeutics	Ataluren	United States	Conducted entirely outside the EU/EEA; registered in relation to a PIP and Article 46
PTC124-GD-046-DMD	2019-001691-11	N/A	NCT03796637	N/A	PTC Therapeutics	Ataluren	United States	Conducted entirely outside the EU/EEA; registered in relation to Article 46
SRP-9001-101	2021-000077-83	N/A	NCT03375164	N/A	Sarepta Therapeutics	Delandistrogene moxeparvovec-rokl	United States	Conducted entirely outside the EU/EEA; registered in relation to a PIP and Article 46
PTC124-GD-045-DMD	2019-001767-67	N/A	NCT03648827	N/A	PTC Therapeutics	Ataluren	United States	Conducted entirely outside the EU/EEA; registered in relation to Article 46
PTC124-GD-048-DMD	2020-000980-21	N/A	NCT04336826	N/A	PTC Therapeutics	Ataluren	United States	Conducted entirely outside the EU/EEA; registered in relation to a PIP and Article 46
R119513	2019-001825-28	N/A	N/A	DMD04	University of Manchester	Lentiviral Vector Trans-Skip gene modified Autologous Mesoangioblasts	United Kingdom	UK-only European trial
GX1001	2017-002213-60	N/A	NCT03368742	IGNITE DMD	Solid Biosciences	SGT-001	United Kingdom	UK-only European trial
WN40226	2015-005455-28	N/A	NCT02515669	Thunderjet	Hoffmann-La Roche	RO7239361 (BMS-986089)	United States, Canada	Conducted entirely outside the EU/EEA; registered in relation to a PIP and Article 46
4658-us-201	2016-005000-26	N/A	NCT01396239	N/A	Sarepta Therapeutics	Eteplirsen	United States	Conducted entirely outside the EU/EEA; registered in relation to a PIP

CZOL446H2337E1	2010-020399-41	N/A	NCT01197300	N/A	Novatris Pharmaceuticals	Zoledronic acid	Australia, Canada, Hungary, Russia, South Africa, United Kingdom	Study population not limited to DMD

Table S3. Investigational medicinal products identified in the included clinical trials and their main characteristics.

IMP	Mechanism of action	Therapeutic group	Trials, n	EU orphan designation	Designation No.
AOC 1044 (delpacibart zotadirsen)	Antibody–PMO conjugate inducing exon 44 skipping	Dystrophin restoration or replacement	1	Active	EU/3/23/2838
Ataluren	Promotes ribosomal read-through of premature stop codons	Dystrophin restoration or replacement	1	Withdrawn	EU/3/05/278
BMN 044	Antisense oligonucleotide inducing exon 44 skipping	Dystrophin restoration or replacement	1	Active	EU/3/08/598
BMN 351	Antisense oligonucleotide inducing exon 51 skipping	Dystrophin restoration or replacement	2	No designation identified	N/A
Casimersen	Phosphorodiamidate morpholino oligomer inducing exon 45 skipping	Dystrophin restoration or replacement	2	No designation identified	N/A
DMD06-MAB	Autologous mesoangioblasts genetically modified ex vivo to induce exon 51 skipping	Dystrophin restoration or replacement	1	No designation identified	N/A

DT-DEC01	Dystrophin-expressing chimeric cell therapy generated by fusion of donor and patient myoblasts	Dystrophin restoration or replacement	1	Active	EU/3/18/2089
DYNE-251	TfR1-targeted Fab–PMO conjugate inducing exon 51 skipping	Dystrophin restoration or replacement	1	No designation identified	N/A
Delandistrogene moxeparvovec	AAV-mediated delivery of a micro-dystrophin transgene	Dystrophin restoration or replacement	5	Active	EU/3/20/2250
ENTR-601-44	Endosomal escape vehicle–PMO conjugate inducing exon 44 skipping	Dystrophin restoration or replacement	2	No designation identified	N/A
ENTR-601-45	Endosomal escape vehicle–PMO conjugate inducing exon 45 skipping	Dystrophin restoration or replacement	2	No designation identified	N/A
Eteplirsen	Phosphorodiamidate morpholino oligomer inducing exon 51 skipping	Dystrophin restoration or replacement	3	Active	EU/3/08/586
Fordadistrogene movaparvovec (PF-06939926)	AAV-mediated delivery of a mini-dystrophin transgene	Dystrophin restoration or replacement	1	Withdrawn	EU/3/16/1716
GNT0004	AAV-mediated delivery of a micro-dystrophin transgene	Dystrophin restoration or replacement	1	Active	EU/3/14/1381
Golodirsen	Phosphorodiamidate morpholino oligomer inducing exon 53 skipping	Dystrophin restoration or replacement	3	No designation identified	N/A

SGT-003	AAV-mediated delivery of a micro-dystrophin transgene	Dystrophin restoration or replacement	2	No designation identified	N/A
SQY51	Antisense oligonucleotide inducing exon 51 skipping	Dystrophin restoration or replacement	1	No designation identified	N/A
Suvodirsen (WVE-210201)	Stereopure antisense oligonucleotide inducing exon 51 skipping	Dystrophin restoration or replacement	3	Active	EU/3/18/2051
Vesleteplirsen (SRP-5051)	Peptide-conjugated PMO inducing exon 51 skipping	Dystrophin restoration or replacement	1	No designation identified	N/A
Viltolarsen	Phosphorodiamidate morpholino oligomer inducing exon 53 skipping	Dystrophin restoration or replacement	3	Active	EU/3/20/2282
Avicursen (ATL1102)	Antisense oligonucleotide targeting CD49d mRNA and reducing VLA-4 expression	Inflammation, fibrosis, immune and epigenetic regulation	1	Active	EU/3/20/2375
Edasalonexent	NF-κB inhibitor with anti-inflammatory and antifibrotic activity	Inflammation, fibrosis, immune and epigenetic regulation	2	Withdrawn	EU/3/15/1560
Givinostat	Histone deacetylase inhibitor with epigenetic and anti-inflammatory effects	Inflammation, fibrosis, immune and epigenetic regulation	4	Active	EU/3/12/1009

MNK-1411	Synthetic ACTH analogue and melanocortin receptor agonist	Inflammation, fibrosis, immune and epigenetic regulation	1	No designation identified	N/A
Pamrevlumab	Monoclonal antibody against connective tissue growth factor with antifibrotic activity	Inflammation, fibrosis, immune and epigenetic regulation	2	Withdrawn	EU/3/19/2234
Satralizumab	Humanised monoclonal antibody against the interleukin-6 receptor	Inflammation, fibrosis, immune and epigenetic regulation	1	No designation identified	N/A
Vamorolone	Dissociative glucocorticoid receptor modulator with anti-inflammatory activity and mineralocorticoid receptor antagonism	Inflammation, fibrosis, immune and epigenetic regulation	7	Active	EU/3/14/1309
BIO101	MAS receptor activator acting through the protective renin–angiotensin system pathway	Muscle protection and regeneration	1	Active	EU/3/18/2030
Domagrozumab (PF-06252616)	Monoclonal antibody inhibiting myostatin	Muscle protection and regeneration	2	Withdrawn	EU/3/13/1105
RO7239361	Anti-myostatin adnectin–Fc fusion protein	Muscle protection and regeneration	1	No designation identified	N/A
SAT-3247 oxalate	AAK1 inhibitor intended to promote muscle stem-cell regeneration	Muscle protection and regeneration	1	No designation identified	N/A

Tamoxifen	Selective estrogen receptor modulator with proposed muscle-protective and antifibrotic effects	Muscle protection and regeneration	1	Active	EU/3/17/1944
Idebenone	Synthetic coenzyme Q10 analogue supporting mitochondrial electron transport and antioxidant activity	Cardiopulmonary function	2	Active	EU/3/07/437
Metoprolol succinate	Selective β1-adrenergic receptor blocker used for cardiac protection	Cardiopulmonary function	1	No designation identified	N/A
Rimeporide	Sodium–hydrogen exchanger-1 inhibitor	Cardiopulmonary function	1	Active	EU/3/15/1478
Imlifidase	IgG-cleaving enzyme used to reduce pre-existing anti-AAV antibodies before gene therapy	Pretreatment; excluded from therapeutic classification	1	No designation identified	N/A
